# Oral Microbiome Transmission and Infant Feeding Habits

**DOI:** 10.1128/mbio.00325-22

**Published:** 2022-04-14

**Authors:** A. M. Kaan, E. Zaura

**Affiliations:** a Department of Preventive Dentistry, Academic Centre for Dentistry Amsterdam (ACTA), Vrije Universiteit Amsterdam and University of Amsterdam, Amsterdam, the Netherlands

**Keywords:** breastfeeding, microbiome, oral, transmission

## Abstract

Transmission of oral microbiota from mother to infant is a highly relevant and, so far, understudied topic due to lack of mainstream high-throughput methods for the assessment of bacterial diversity at a strain level. In their recent article in *mBio*, S. Kageyama, M. Furuta, T. Takeshita, J. Ma, et al. (mBio 13:e03452-21, 2021, https://doi.org/10.1128/mbio.03452-21) evaluated oral microbial transmission from mothers to their infants by using full-length analysis of the 16S rRNA gene and demonstrated the applicability of this method for assessment of transmission of oral bacteria at the single-nucleotide-difference level. By analyzing different metadata of the mother-infant pairs, they discovered that the presence of maternal oral bacteria was higher in formula-fed infants compared to infants who were breastfed or received mixed feeding. This interesting finding suggests that breastfeeding may prevent early maturation of infant’s oral microbiome. The physiological role of this phenomenon still needs to be elucidated.

## COMMENTARY

## DETECTION OF STRAIN-LEVEL DIVERSITY OF HUMAN MICROBIOME

The human microbiome is shaped by an interplay of factors, including genetic background, immune system, culture, economics, behavior, and environment (1). It is acquired and developed in the first years of life and is thought to play a critical role in the immune, endocrine, metabolic, and other developmental pathways of the child (2). Therefore, understanding of the process of acquisition of the microbiome and steering this process toward the most optimal health benefit are of utmost importance.

Although the maternal microbiome is a major source of one’s microbes early in life, mother-infant microbiome transmission is an understudied topic. This lack of knowledge could be due to the lack of high-throughput approaches for distinguishing microbes at a strain level. For decades, strain-level diversity of bacteria from different clinical samples has been assessed by culture-based and low-throughput molecular methods on individual isolates (3).

Developments in high-throughput sequencing technologies have led to major advances in the field. Currently, the ultimate method for strain-level resolution is metagenomic or shotgun sequencing (4, 5). This method, unlike 16S rRNA gene fragment amplicon sequencing, does not require PCR amplification and provides information on functional genes of all members of the microbial community. This way, transmission of gut bacterial strains from mother to child was assessed longitudinally in the first months of life of 44 infants (4). The authors discovered two different strain inheritance patterns: transmission of the mother’s dominant strains and transmission of the secondary strains. Based on their functional genes, the secondary strains appeared to have a selective advantage in the infant gut compared to the dominant strains. For the oral cavity, a metagenomic analysis of dental plaque samples from 30 children with and without dental caries provided strain-specific associations with tooth decay for several species (5). However, metagenomic sequencing is time-consuming and requires high computational resources.

The mainstream method for microbial community profiling—16S rRNA gene fragment amplicon sequencing—usually covers one to two hypervariable regions (100 to 500 bp) of this marker gene. The taxonomic resolution of these short reads is not always informative enough at the species level, but certainly not at the strain level. In recent years, long-read sequencing (tens of thousands of base pairs) technologies have been developed by Pacific Biosciences (PacBio) and Oxford Nanopore. The high error rate of long sequencing reads (∼10%) compared to short reads (∼0.5%) can now effectively be ameliorated by the construction of circular consensus sequences (PacBio) combined with data denoising algorithms (DADA2) (6). This method now allows full-length (∼1,500-bp) sequencing of the 16S rRNA gene at a single-nucleotide resolution. This way, long-read sequencing was shown to be able to resolve divergent copies of the 16S rRNA gene existing within the same genome, thereby improving discrimination between species and even strains in 16S gene-based microbiome studies (7). Kageyama et al. (8) have elegantly applied this approach to assess acquisition of oral microbiota from mother to child in 448 mother-infant pairs. They found that infants shared 0 to 40 amplicon sequence variants (ASVs) with their mother, accounting for 0 to 99.3% (median, 9.3%) of the total abundance of their microbiota.

Some oral species, but especially strains of the same species, can be highly homologous at the 16S rRNA gene level. This is particularly the case for Streptococcus oralis and Streptococcus mitis, due to their high evolutionary relatedness but also due to lateral 16S rRNA gene transfer between these species (9). Since these are also highly abundant taxa in infant oral microbiome, strain-level determination using the 16S rRNA gene alone most likely underestimates their diversity. Combining the 16S rRNA gene data with the intergenic spacer region (ISR) of 16S to 23S has been shown to provide better strain-level resolution than the 16S rRNA gene alone (10, 11). This seems to be a promising strategy, especially if it could be combined with a long-read sequencing approach.

### Infant feeding habits and microbial transmission.

An unexpected and very interesting finding of the study by Kageyama et al. (8) was the greater acquisition of maternal oral bacteria in formula-fed infants compared to breastfed and mixed-fed infants. Interindividual differences in microbial colonization depend on many factors, including microbial load in saliva of mothers, frequency of contact, nutrient sources, and the receptiveness of the oral environment of the child (12).

The results of Kageyama et al. suggest that breastfeeding is associated with delayed microbial maturity, which is in line with findings on gut microbiome of infants (13). The health effects of delayed maturation of oral microbiome have not been addressed yet, while for the gut a temporary high microbial maturity very early in life was associated with an increased risk of allergic sensitization and asthma at school age (13). The authors of the latter study explained their findings with a dysregulated colonization process in which some taxa might be arriving too early rather than having an overall more mature community structure. Among the oral bacterial species that were significantly enriched in formula-fed infants of the study by Kageyama et al. were Prevotella melaninogenica, Granulicatella adiacens, Veillonella dispar, and Schaalia odontolytica (former Actinomyces odontolyticus) (8). It remains to be investigated if a higher relative abundance in the oral cavity of these and other taxa early in life has health consequences at a later age.

The mechanisms for the observed differences in microbial acquisition by feeding habits are not clear yet. It is likely that breast milk with its immunological and antimicrobial components reduces certain microbial colonization in the oral cavity. On the other hand, habitual procedures during the formula feeding, such as licking the nipple of the bottle before feeding to test the temperature, should be considered as well.

Maternal oral health is shown to influence bacterial transmission from mother to infant (14, 15), where poor oral health usually comes with higher microbial load and more frequent microbial transfer. The eruption and presence of first teeth for the infant are another important factor for microbial colonization. This process creates a unique, nonshedding surface for microbial attachment and growth, leading to an increase in microbial diversity. Future studies on oral strain transmission and colonization should therefore include the oral health status of both the mother and infant.

The study by Kageyama et al. (8) raises several questions for further research. It is to be discerned how the rate of microbial maturation at an early age affects child’s health at a later age. It is possible that certain untimely acquired microbiota elicit a different immune response and affect the maturation of the immune system and thereby the health of the child.
